# High-Quality Draft Genome Sequences of the Uncultured Delta3 Endosymbiont (Deltaproteobacteria) Assembled from Metagenomes of the Gutless Marine Worm Olavius algarvensis

**DOI:** 10.1128/MRA.00704-20

**Published:** 2020-07-30

**Authors:** Yui Sato, Juliane Wippler, Cecilia Wentrup, Tanja Woyke, Nicole Dubilier, Manuel Kleiner

**Affiliations:** a Max Planck Institute for Marine Microbiology, Bremen, Germany; b University of Vienna, Department of Microbiology and Ecosystem Science, Vienna, Austria; c DOE Joint Genome Institute, Berkeley, California, USA; d University of Bremen, Faculty 2 Biology/Chemistry, Bremen, Germany; e North Carolina State University, Department of Plant and Microbial Biology, Raleigh, North Carolina, USA; Georgia Institute of Technology

## Abstract

Here, we present two high-quality, draft metagenome-assembled genomes of deltaproteobacterial OalgDelta3 endosymbionts from the gutless marine worm Olavius algarvensis. Their 16S rRNA gene sequences share 98% identity with Delta3 endosymbionts of related host species *Olavius ilvae* (GenBank accession no. AJ620501) and *Inanidrilus exumae* (GenBank accession no. FM202060), for which no symbiont genomes are available.

## ANNOUNCEMENT

Gutless phallodriline annelids are a species-rich, monophyletic group of marine oligochaetes that lack a digestive system (1). Instead, they rely on phylogenetically diverse bacterial symbionts that provide them with nutrition via chemosynthesis (2–5). Symbionts of different host species show host specificity but often form closely related bacterial clades. To date, none of the symbionts has been cultivated, but comparative genomic analyses have been performed using high-throughput metagenomic sequencing (3, 6). Here, we present two draft genomes of a deltaproteobacterial symbiont from the host Olavius algarvensis, termed OalgDelta3. OalgDelta3 is closely related to uncultured Delta3 endosymbionts from two other gutless phallodrilinids (6, 7), namely, *Olavius ilvae* and *Inanidrilus exumae*, sharing 98% sequence identity with their 16S rRNA genes (GenBank accession no. AJ620501 and FM202060, respectively), and to deltaproteobacteria (SEEP-SRB1) that form consortia with anaerobic methane oxidizers in anoxic marine sediments (8, 9).

Two O. algarvensis specimens were collected from Elba, Italy (specimen B2SA from Sant’Andrea [42°48'26.00"N, 10°8'28.00"E] in April 2013 and specimen A1CA from Cavoli [42°44'4.15"N, 10°11'10.47"E] in June 2014), as described previously (4), and stored at 4°C in RNAlater (Ambion Life Technologies). DNA was extracted from individual specimens using the Qiagen blood and tissue kit. Two separate DNA libraries were prepared from mechanically sheared (Covaris LE220), size-selected (using Beckman Coulter solid-phase reversible immobilization beads targeting 300 bp) total DNA using the KAPA library creation kit for Illumina platforms (Kapa Biosystems) and underwent paired-end sequencing on the Illumina HiSeq 2000 platform (2 × 150 bp) at the DOE Joint Genome Institute. A total of 53,614,071 and 36,269,211 raw read pairs were obtained from B2SA and A1CA, respectively.

Reads were quality controlled with BBDuk (minimum kmer, 11; minimum length, 36 bp; minimum Phred quality score, 2) of the BBMap toolkit v36.86 (https://sourceforge.net/projects/bbmap) and BayesHammer implemented in SPAdes v3.9.1 (10, 11), and 53,474,255 (B2SA) and 36,195,816 (A1CA) clean read pairs were separately assembled with MEGAHIT v1.0.6 (12). Metagenome-assembled genomes (MAGs) were obtained using MetaBAT v0.26.3 (13) and refined using Bandage v0.08.1 (14). Genome completeness and contamination were estimated using CheckM v1.0.5 (15). Average nucleotide identity (ANI) was calculated with enveomics tools (16). Genome annotation and analysis were performed with RASTtk (17–19) and Pathway Tools v21.0 (20). Default parameters were used for all software unless otherwise noted.

MAGs were assigned as OalgDelta3 by identifying the previously reported 16S rRNA gene sequence (GenBank accession no. AM493254) with >99.5% identity. The OalgDelta3 MAG from B2SA has 5.58 Mbp (401 contigs, with an *N*_50_ value of 76,958 bp and the largest contig of 424,721 bp; GC content, 54.2%; genome coverage, 50×) and contains 5,795 protein-coding sequences (CDSs) and 43 tRNA-coding regions. The OalgDelta3 MAG from A1CA has 5.74 Mbp (350 contigs, with an *N*_50_ value of 89,163 bp and the largest contig of 348,870 bp; GC content, 54.1%; genome coverage, 350×) and includes 5,876 CDSs and 45 tRNA-coding regions. The two MAGs share 99.86% identical 16S rRNA gene sequences, with a genome-wide ANI of 99.87%. Both MAGs have 94.4% estimated completeness, with no (B2SA) or very low (A1CA; 0.03%) contamination estimates, and conform to the MIMAG standards for high-quality draft genomes (21). Similar to other deltaproteobacterial symbionts of O. algarvensis, OalgDelta3 harbors pathways for sulfate reduction, carbon monoxide and H_2_ oxidation, utilization of sugars, short-chain fatty acids and oligopeptides, and a multitude of associated ATP-binding cassette (ABC) transporters, tripartite ATP-independent periplasmic (TRAP)-type transporters, and other transporters.

### Data availability.

The MAGs of the OalgDelta3 endosymbiont have been deposited in the European Nucleotide Archive under accession no. PRJEB28157 (assemblies GCA_903231395 and GCA_903231505), using the data brokerage service of the German Federation for Biological Data (GFBio) (22), in compliance with the Minimal Information about any (x) Sequence (MIxS) standard (23). The raw sequences are available at the Sequence Read Archive (SRA) under accession no. SRX2712534 and SRX2554373.
